# Codon usage pattern of the ancestor of green plants revealed through Rhodophyta

**DOI:** 10.1186/s12864-023-09586-w

**Published:** 2023-09-11

**Authors:** Huipeng Yao, Tingting Li, Zheng Ma, Xiyuan Wang, Lixiao Xu, Yuxin Zhang, Yi Cai, Zizhong Tang

**Affiliations:** College of Life Science, Sichuan Agriculture University, Ya’an, 625014 Sichuan People’s Republic of China

**Keywords:** Rhodophyta, Optimal codons, Codon usage bias, Translational accuracy, tRNA modification

## Abstract

Rhodophyta are among the closest known relatives of green plants. Studying the codons of their genomes can help us understand the codon usage pattern and characteristics of the ancestor of green plants. By studying the codon usage pattern of all available red algae, it was found that although there are some differences among species, high-bias genes in most red algae prefer codons ending with GC. Correlation analysis, Nc-GC3s plots, parity rule 2 plots, neutrality plot analysis, differential protein region analysis and comparison of the nucleotide content of introns and flanking sequences showed that the bias phenomenon is likely to be influenced by local mutation pressure and natural selection, the latter of which is the dominant factor in terms of translation accuracy and efficiency. It is worth noting that selection on translation accuracy could even be detected in the low-bias genes of individual species. In addition, we identified 15 common optimal codons in seven red algae except for *G. sulphuraria* for the first time, most of which were found to be complementary and bound to the tRNA genes with the highest copy number. Interestingly, tRNA modification was found for the highly degenerate amino acids of all multicellular red algae and individual unicellular red algae, which indicates that highly biased genes tend to use modified tRNA in translation. Our research not only lays a foundation for exploring the characteristics of codon usage of the red algae as green plant ancestors, but will also facilitate the design and performance of transgenic work in some economic red algae in the future.

**Supplementary Information:**

The online version contains supplementary material available at 10.1186/s12864-023-09586-w.

## Introduction

**Fig. 1:**
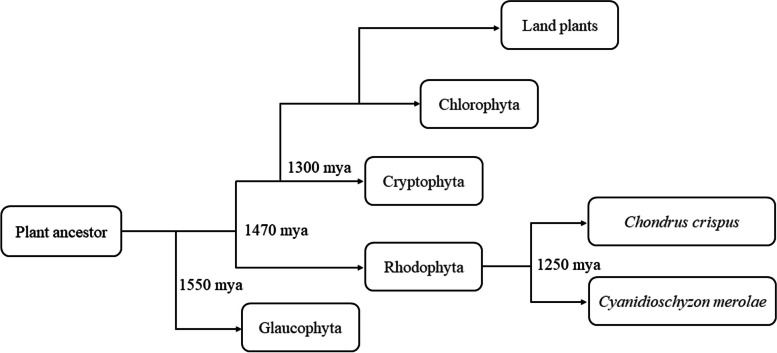
Simplified phylogeny of plant evolution. Mya represents millions of years ago. Approximate divergence dates are taken from Parfrey*, *et al*.* [14]. *Cyanidioschyzon merolae* and *Chondrus crispus* are representatives of unicellular and multicellular red algae respectively

Plants in a broad sense, known as Archaeplastida, are a large and diverse group, including green plants (Viridiplantae), red algae (Rhodophyta) and glaucophytes (Glaucophyta), all of which can carry out photosynthesis in their chloroplasts evolved from cyanobacteria through the primary endosymbiosis process [1–6]. Some fossil [7–10], molecular clock [11–15] and phylogenetic studies [16–19] have shown that Rhodophyta appeared approximately 1.5 billion years ago and may be the closest ancestors of Viridiplantae (Fig. 1).

Codons are the bridge between genes and proteins. In a genome, the phenomenon of unequal usage of synonymous codons is called codon bias, which exists widely in different species [20]. The level of codon usage bias in a gene is commonly determined using the value of Nc (effective number of codons) ranging from 20 to 61, which reflects the degree of preference for codon usage [21]. A large number of researchers have proposed that natural selection, mutation pressure and even genetic drift can affect codon usage patterns in species [22–24]. In some species, natural selection is the main factor driving codon usage, and if they have a set of optimal codons for translation, it will provide them with a selective advantage over species with other codons under selection pressure [24, 25]. Because the frequency of codon usage matched the cellular tRNA population, Ikemura [25] suggested that the optimal codon matched the most abundant isoaccepting tRNA and the optimal codons were generally frequently used in highly expressed genes [25, 26].

Suzuki*, *et al*.* [27] studied the codon adaptation of plastid genomes of 12 red algae, and found that the relative strength of selection varied greatly in the Rhodophyta, with some showing strong bias, and others showing weaker bias. Li*, *et al*.* [28] analyzed the codon usage pattern of the chloroplast genome of *Porphyra umbilicalis* and found that it was mainly affected by natural selection, mutation pressure and nucleotide composition. Lee*, *et al*.* [29] studied expressed sequence tags (ESTs) to analyze codon usage in *Griffithsia okiensis*, *Chondrus crispus* and *Porphyra yezoensis* and found that they had higher GC-content bias and that most of the optimal codons of *Griffithsia okiensis* as well as *Chondrus crispus* ended with C, but that of *Porphyra yezoensis* ended with G. However, there have been few studies on the codon patterns related to the nuclear genome of red algae.

In this study, we performed a detailed analysis of codon usage patterns, influencing factors, optimal codons and corresponding tRNA genes of all nuclear coding genes in four unicellular Rhodophyta and four multicellular Rhodophyta, which might not only lay a foundation for exploring the characteristics of codon usage of red algae as green plant ancestors, but also will aid in the design and performance of transgenic work in some economic red algae to maximize corresponding protein yield in the future.

## Results

### Direction and strength of codon usage bias in the rhodophyta

The size of the genome and the number of CDSs extracted from eight red algae (*C. merolae, G. sulphuraria, C. yangmingshanensis, C. crispus, G. chorda, G. domingensis, P. purpureum* and *P. umbilicalis*) are presented in Table 1, showing that they are 16–23 Mb and 4803–9898 in unicellular red algae and 78–105 Mb and 9603–13360 in multicellular species, respectively. The effective number of codons (Nc) can be used to evaluate the degree of codon usage bias. According to Table 1, the Nc values ranged from 40 to 56 in these species, in which *P. umbilicalis* shows the strongest codon usage bias, followed by *G. sulphuraria, P. purpureum* and *C. crispus.* In addition, the codon usage biases of the other four species were similar.

**Table 1:** Whole genome and codon usage statistics in the coding sequence of eight red algae

Species	Genome Size (Mb)	Number of CDS	Number of CDS (after filtering)	GC-Content	GC3s (± SD)	GC3 (± SD)	Nc (± SD)	Fop (± SD)	$\widehat{s}$
*C. merolae*	16.55	4803	4713	0.550	0.581 ± 0.046	0.594 ± 0.045	55.29 ± 3.39	0.470 ± 0.032	0.61
*G. sulphuraria*	13.71	7174	5960	0.379	0.313 ± 0.049	0.337 ± 0.047	49.81 ± 4.26	0.367 ± 0.037	0.97
*C. yangmingshanensis*	12.07	5185	5174	0.546	0.554 ± 0.048	0.569 ± 0.047	55.88 ± 3.41	0.447 ± 0.034	0.39
*P. purpureum*	22.19	9898	9611	0.559	0.635 ± 0.073	0.648 ± 0.070	51.54 ± 5.32	0.466 ± 0.038	1.21
*C. crispus*	104.8	9607	7133	0.529	0.585 ± 0.119	0.599 ± 0.115	52.98 ± 7.41	0.440 ± 0.050	1.48
*G. chorda*	92.18	10,813	10,021	0.493	0.504 ± 0.087	0.521 ± 0.084	55.29 ± 5.17	0.444 ± 0.046	0.68
*G. domingensis*	77.95	11,532	10,869	0.497	0.505 ± 0.099	0.520 ± 0.095	54.72 ± 5.62	0.444 ± 0.056	0.77
*P. umbilicalis*	87.7	13,332	12,044	0.658	0.816 ± 0.084	0.820 ± 0.082	40.27 ± 6.06	0.495 ± 0.045	1.42

*Mb* represents megabases, *CDS* represents coding sequence, *GC3s* represents the guanine + cytosine content of the silent 3rd codon position, *Nc* represents the effective number of codons, *Fop* represents the frequency of optimal codons, *Ŝ* represents the selection strength of codon usage bias. Genome size (Mb), number of CDS, and GC-content were all taken from the NCBI database(https://www.ncbi.nlm.nih.gov/). Number of CDS after filtering is the final number of CDS that meets all filtering criteria

The G or C nucleotide content of the third synonymous position (GC3s) can indicate the bias direction of the synonymous codon of a species or a gene. According to Table 1, *G. sulphuraria* is the only species that exhibits poor GC bias. The GC3s content of the CDSs was only approximately 0.313 ± 0.049(mean ± SD). The GC3s contents of *G. chorda* and *G. domingensis* were medium at approximately 0.5, specifically 0.504 ± 0.087 and 0.505 ± 0.099, respectively. Contrary to *G. sulphuraria*, there was strong GC bias across the other five red algae, in which *P. umbilicalis* had the highest GC3s (0.816 ± 0.084), followed by *P. purpureum* (0.635 ± 0.073).

In short, *P. purpureum* and *P. umbilicalis* show a strong preference for codons ending with GC. *C. yangmingshanensis, C. merolae* and *C. crispu*s show a preference for codons ending with GC, but this preference is not strong. *G. chorda* and *G. domingensis* almost show a neutral preference for four codons. *G. sulphuraria* is different from the other species and strongly prefers codons ending with AT. Therefore, there are different codon usage biases among different red algae.

### The role of mutation pressure in codon usage

**Fig. 2:**
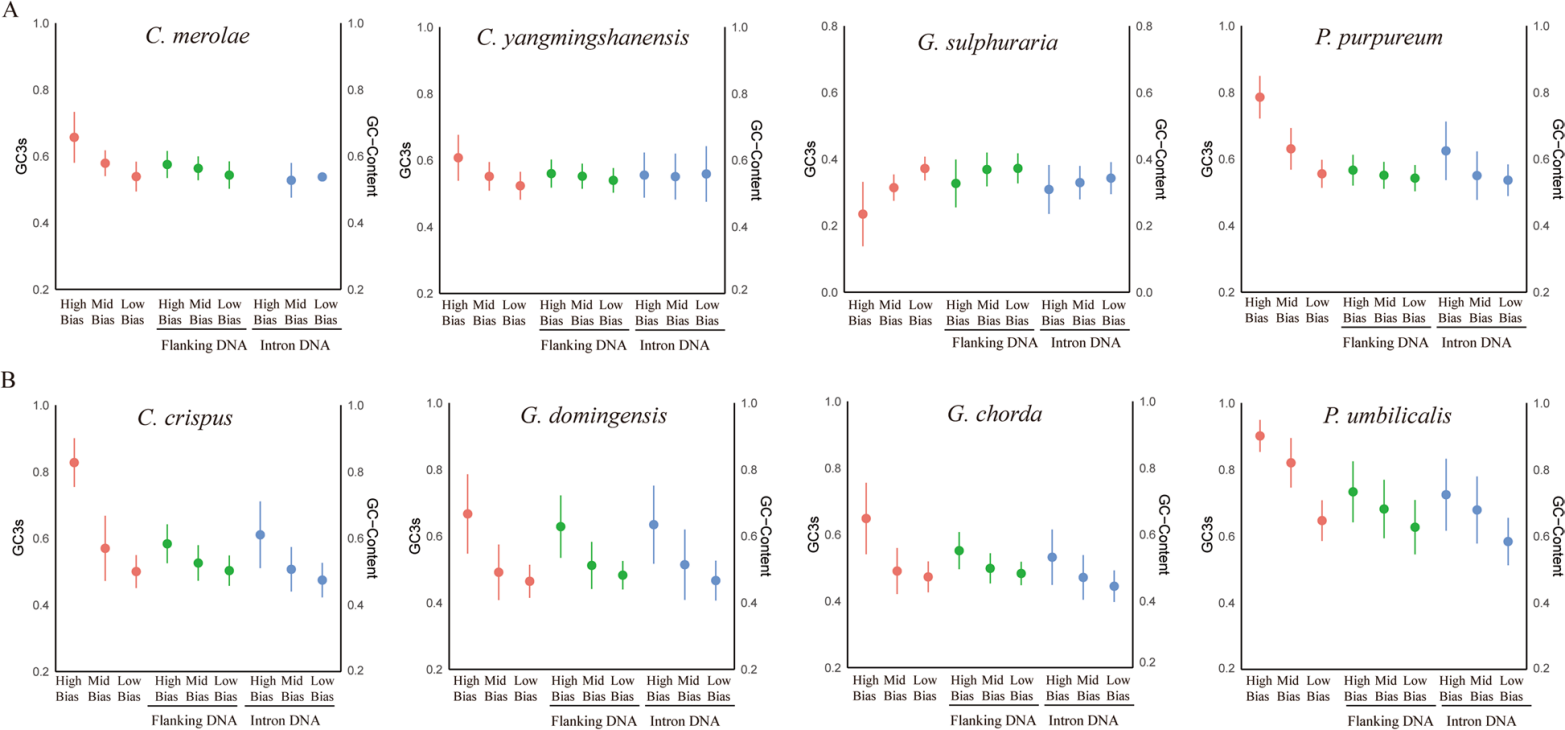
Comparison of the average GC3s for coding sequences and GC content for noncoding DNA in red algae. The line chart represents the standard deviation of each category value. Red dots represent the value of GC3s shown on the left y-axis (from left to right: high-bias genes, mid-bias genes and low-bias genes). The GC of noncoding is displayed on the right y-axis (flanking DNA: green dots; intron DNA: blue dots. From left to right: high-bias genes, mid-bias genes and low-bias genes). **A**: unicellular red algal group; **B**: multicellular red algal group

To confirm the influence of mutation pressure on the codon usage pattern in plant ancestor species, we calculated the GC3s of coding sequences, as well as the GC of introns and flanking DNA in high-bias, medium-biased and low-bias gene categories from eight red algae genomes. The results are shown in Fig. 2 and Supplementary Table S1. High-bias genes in the seven red algae preferred codons ending with GC, except for those in *G. sulphuraria*. In addition, the GC3s of the coding sequences and GC of flanking DNA and introns decreased with the decreasing of preference level in the seven species except for *G. sulphuraria*. In other words, both GC3s and GC were the highest in the high-bias genes, while they were the lowest in the low-bias genes. However, the GC patter of introns was relatively complex. For four multicellular species, the variation trend of the GC content of introns was consistent with that of the codon bias. However, it was different from that in the four unicellular species. For example, the GC content of the introns in *P. purpureum* was the highest among the high-bias genes, but the lowest in *G. sulphuraria* and remained relatively stable in *C. yangmingshanensis*. By pairwise comparison in three different bias categories (high bias and mid bias, mid bias and low bias) for eight species, it was found that the difference was very significant in most comparisons (Supplementary Table S1). In particular, there was no intron in the high-bias genes and only one intron in the low-bias genes in *C. merolae.* Therefore, it was impossible to carry out t tests between different categories in *C. merolae* (Supplementary Table S1). Additionally, except for *G. domingensis*, the GC3s in the mid-bias genes was similar that in the low bias genes, which was the same as the content of GC in the noncoding regions among the three bias categories (Supplementary Table S1). However, in the high-bias gene category, GC3s was markedly different from the content of GC, which indicates that natural selection plays a dominant role in codon usage across the high-bias gene category. Although the GC3s of CDSs was not related to the GC content of introns in unicellular red algae, a strong correlation was found in multicellular red algae (Supplementary Figure S1 and Supplementary Table S2). Additionally, the stop codons showed a preference for UAA over both UAG and UGA with G in highly biased genes of all seven species (Supplementary Table S3), which proves that the GC bias was not caused mainly by mutation pressure.

Overall, mutation pressure exerts a limited influence, so it is supposed that natural selection strongly affects the codon usage pattern of red algae by driving the high GC content of highly biased genes, especially in unicellular species.

### Nc-GC3s plot

**Fig. 3:**
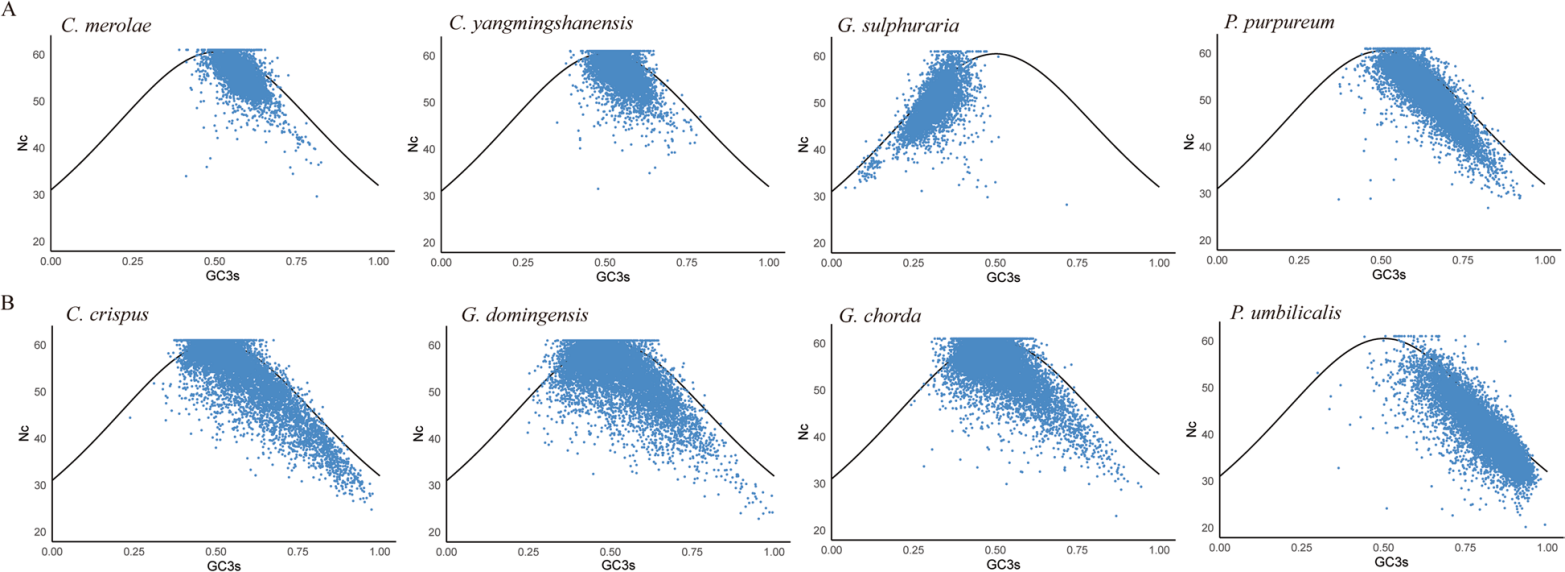
The relationship between the effective number of codons (Nc) and GC content at the third synonymous codon position (GC3s) in eight red algae. In each plot, the curved line represents the expected position of genes under a neutral mutation model. **A**: unicellular red algal group; **B**: multicellular red algal group

The GC3s content of each gene in the genome of red algae was taken as the abscissa, and Nc was taken as the ordinate (Fig. 3). In the plot, only some of the actual observed values of each red algal gene are very close to the expected values, indicating that the codon use of these genes is almost entirely caused by mutation, and most of the gene loci are located at a position deviating from the expected curve, indicating that the codon preference of red algal genes is more affected by selection.

### Neutrality plot analysis

**Fig. 4:**
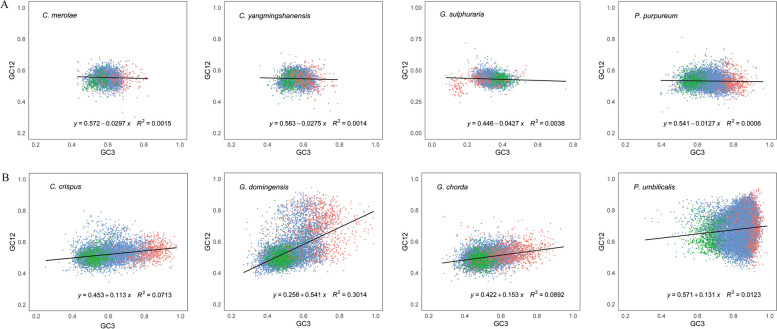
Neutrality plot analysis of eight red algae between GC12 (the mean GC content at the first and second positions) and GC3 (GC content at the third codon position). The black solid line represents the correlation line, and the equation of the correlation line is shown below the plot. The red dots represent the high-bias genes, the blue dots represent the medium-bias genes, and the green dots represent the low-bias genes. **A**: unicellular red algal group; **B**: multicellular red algal group

To determine the main influencing factors of codon usage preference of the whole genome of red algae, neutrality plot analysis of the coding sequences of eight red algae was performed, as shown in Fig. 4. The slope of the plots shows the proportion of mutation pressure influencing the codon usage pattern. According to Fig. 4, mutation pressure accounts for less than 50% across all red algae, except *G. domingensis*(54.1%)*.* For the four unicellular red algae, the slope is not more than 5% (*C. merolae*: 2.97%, *C. yangmingshanensis*: 2.75%, *G. sulphuraria*: 4.27% and *P. purpureum*: 1.27%) which indicates that natural selection and other factors play a dominant role in the formation of codon usage patterns. Similarly, in the four multicellular red algae (*C. crispus, G. chorda, P. umbilicalis* and *G. domingensis*), natural selection and other factors accounted for 74.2%, 84.7%, 86.9% and 45.9%, respectively. Moreover, as shown in Supplementary Table S7, GC12 and GC3 showed weak significant correlations in the six red algae (all *r* < 0.4, *P* < 0.01), strong significant correlations in *G. domingensis* (*r* = 0.730, *P* < 0.01) and general correlations in *P. umbilicalis* (*r* = 0.569, *P* < 0.01). In summary, mutation pressure may drive the codon usage bias of *G. domingensis* more strongly*,* while natural selection plays a leading role in the formation of codon usage patterns among the other seven red algae.

### Parity rule 2 plot

**Fig. 5:**
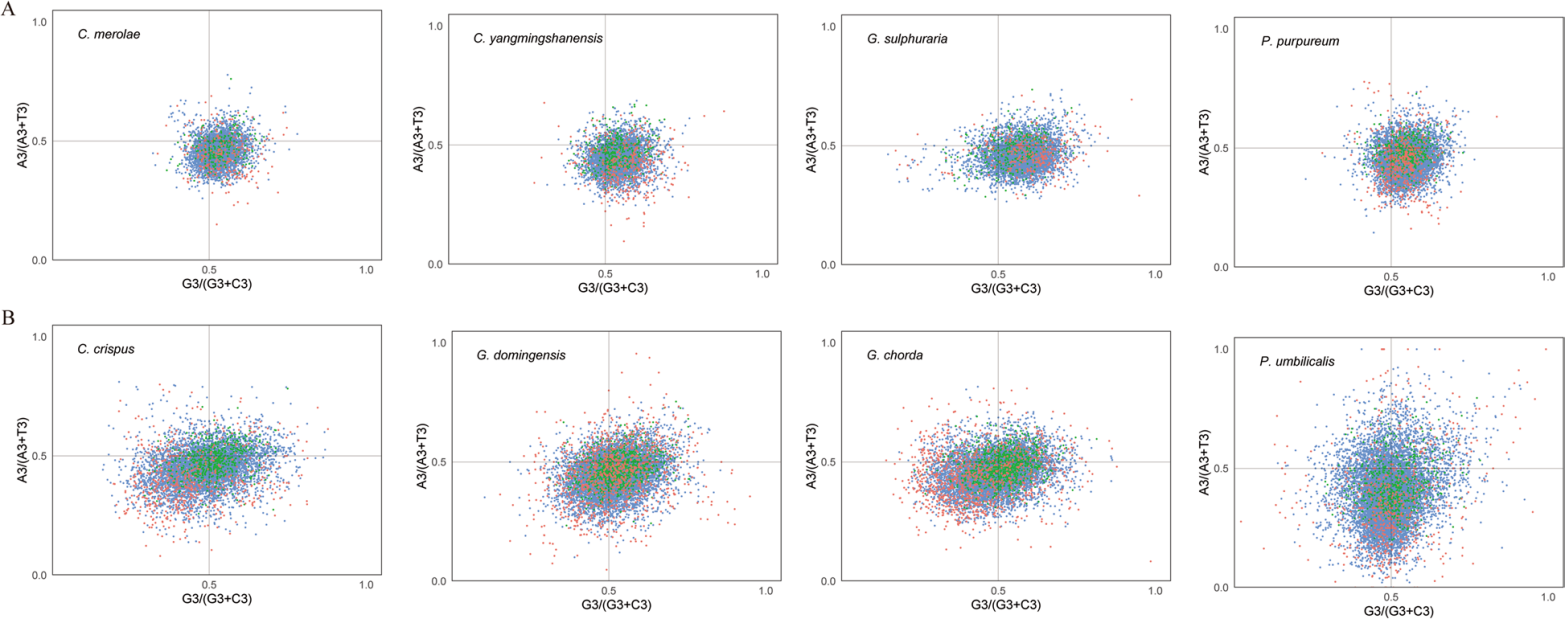
Parity rule 2 plot analysis of eight red algae (GC bias on the x-axis and AT bias on that y-axis). The center point at 0.5 represents A = T and G = C, which means that there is no codon usage deviation between the two DNA strands. The red dots represent the high-bias genes, the blue dots represent the medium-bias genes, and the green dots represent the low-bias genes. **A**: unicellular red algal group; **B**: multicellular red algal group

The third base of the codon is closely related to the formation of codon usage preference. Parity rule analysis (PR2) of all genes across the 8 red algae is shown in Fig. 5, revealing that the points representing genes are unevenly distributed in four regions. Most of the points for the four unicellular red algae are scattered in the fourth quadrant (the ratio of A3/(A3 + T3) < 0.5 and G3/(G3 + C3) > 0.5) while the fewest are found in the first quadrant, which shows that at the third base of the codon, T is used more frequently than A, and G is used more frequently than C. In three multicellular red algae, most of the points are scattered in the third quadrant (the ratio of A3/(A3 + T3) < 0.5 and G3/(G3 + C3) < 0.5), illustrating that T and G are used more frequently for the third base. However, for *G. domingensis,* the number of points in the fourth quadrant was slightly greater than that in the third quadrant. In short, the third base of the codons shows a preference for G, C and T across all eight species.

### Identification of optimal codons and major trna genes

Optimal codons can also be used to determine the influence strength of natural selection on codon bias. The results of the optimal codons for all 8 red algae are shown in Table 2 and Supplementary Table S3, from which it is seen the optimal codons of *G. sulphuraria* is end in A and U, except for one (UUG) ending in G. For the other seven species, 163 of 165 optimal codons end in G or C, except for CGU and GGU ending in uracil for *C. yangmingshanensis*. Furthermore, CGU and GGU are used with the lowest frequency in these optimal codons of arginine and glycine respectively (Supplementary Table S3). Significantly, there was no optimal codon ending in adenine for the seven species. Among the 18 amino acids including synonymous codons, phenylalanine is the only amino acid without an optimal codon in *C. yangmingshanensis*. Nine twofold degenerate amino acids have the same optimal codons, and seven of the nine threefold, fourfold and sixfold degenerate amino acids (except for valine and threonine) share at least one optimal codon, which indicates that the characteristics of optimal codons are significantly similar across all seven species.

**Table 2:**
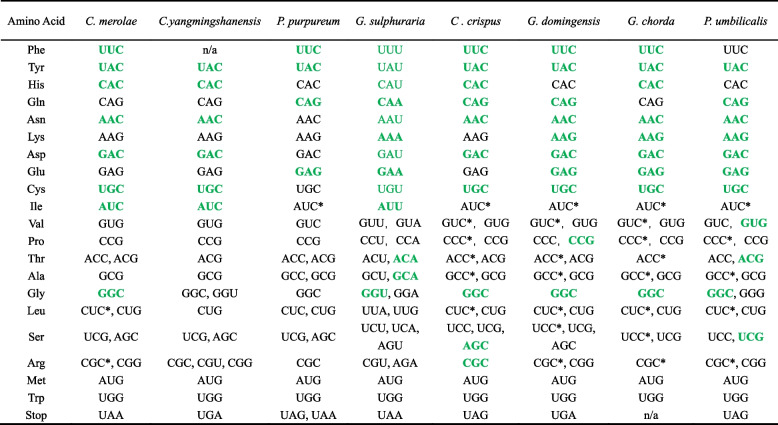
Optimal codons designated for the eight species of red algae

The optimal codons that are complementary to the major tRNA genes are rewritten in green; an asterisk indicates the optimal codon that complementarily matches the major tRNA gene with the deaminated adenosine at the wobble site; the optimal codons that are complementary to the major tRNA genes with G complementary to uracil in the wobble position are written in green normal font

tRNA genes were identified to compare their optimal codons with those of major tRNA genes for all species. Major tRNA genes were defined as the most abundant tRNA gene for one amino acid. The tRNA genes of all eight species are shown as Supplementary Table S4, indicating that the copy number of tRNA genes was higher in multicellular species (97–165) than in unicellular red algae (22–26), except for *G. sulphuraria* (82). However, a few tRNA genes, such as tRNA ^Ile^, appeared only in the unicellular species *C. merolae* and *C. yangmingshanensis*.

Except for methionine and tryptophan, 18 common amino acids encoded by 59 synonymous codons included nine twofold degenerate amino acids, one threefold degenerate amino acid, five fourfold degenerate amino acids and three sixfold degenerate amino acids. For the nine twofold degenerate amino acids, all major tRNA genes matched their optimal codons in *G. sulphuraria* (including 6 G-U matches) and most major tRNA genes corresponding to optimal codons (47/62) were also identified in the other 7 species (Table 2 and Supplementary Table S6). However, for threefold, fourfold and sixfold degenerate amino acids, there were only thirteen matches between optimal codons and the major tRNA genes in the seven species other than *G. sulphuraria*, in which the common match of glycine (GGC/GCC) simultaneously appeared in the six species other than *G. sulphuraria* and *P. purpureum* (Table 2). However, in most major tRNA genes in multicellular red algae (26/32), there were many bases of adenine at the anticodon position (Supplementary Tables S4, S5 and S6). It is inferred that these adenines are deaminated into inosine to pair with the cytosine of the optimal codon by the wobble effect.

Because adenine deamination appears rarely in unicellular red algae, the following analysis was performed on multicellular red algae. Among the six optimal codons perfectly matching major tRNA genes, CCG was complementary to the major tRNA genes of proline (GGC) in *G. domingensis*. AGC and CGC were complementary to the major tRNA genes of serine (GCU) and arginine (GCG) in *C. crispus*. GUG, ACG and UCG were complementary to the major tRNA genes of valine (CAC), threonine (CGU) and serine (CGA) in *P. umbilicalis* (Table 2, Supplementary Tables S4, S5 and S6)*.* Except for these codons, for threefold to sixfold-degenerate amino acids, it was found that other optimal codons ended with cytosine. The matching major tRNA genes had adenine at the wobble site (Table 2 and Supplementary Tables S4, S5 and S6), which indicates that in the process of tRNA gene evolution, adenosines at the sites were deaminated to inosines to match the cytosine at the degenerate position. Overall, the major tRNA genes of most amino acids always matched their most frequently optimal codons in these species.

Surprisingly, using tRNAscan-SE software, the tRNASeC gene, the tRNA gene for selenocysteine (anticodon: TCA), was found to pair with the UGA codon in all four unicellular red algae but not in the four multicellular red algae (Supplementary Tables S4 and S6).

### Correlation analysis

The expression of genes can be roughly assessed through the codon bias parameters, i.e., Fop (frequency of optimal codons), the CAI (codon adaptation index) and the CBI (codon bias index) [30–33]. Correlation analysis of all codon bias indicators of the protein-coding genes in all eight red algae is shown in Supplementary Table S7, showing that Nc was significantly correlated with Fop, the CAI and the CBI in all species. The correlation coefficient r was lower than 0.55(most r = 0.4–0.55, few r < 0.4) which indicates that the gene expression level may have some influence on the codon usage pattern of 8 species.

### Protein domain codons show stronger codon preferences than nondomain codons

**Fig. 6:**
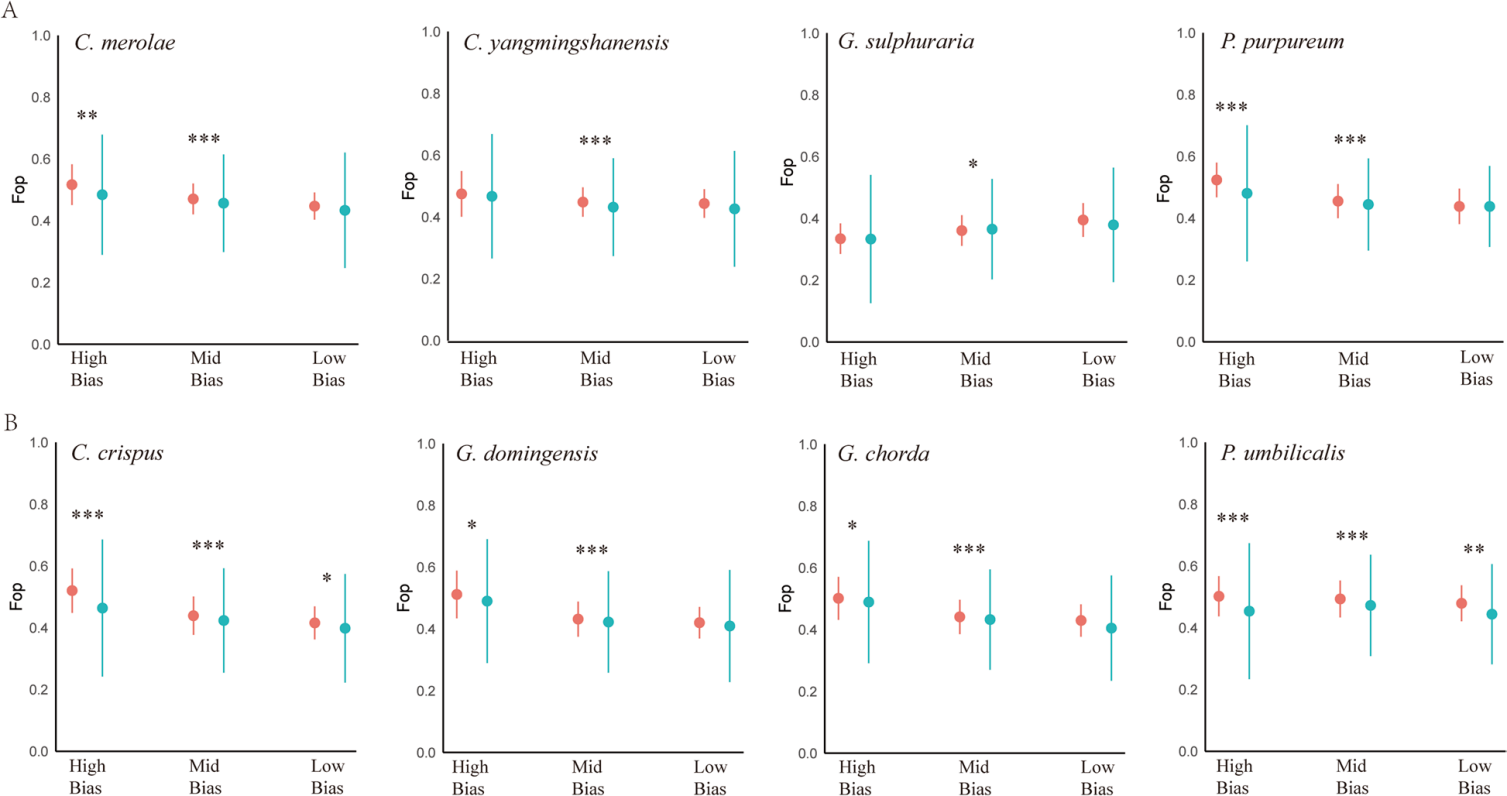
Average Fop value in domain codons and nondomain sequence codons. The red dots represent the average values of domain codons, and the blue-green dots represent the average values of nondomain codons. The line graph shows the standard deviation of each bias category value (*** *P* < 0.001; **P < 0.01; *P < 0.05). From left to right: high-bias genes, medium-bias genes and low-bias genes. **A**: unicellular red algal group; **B**: multicellular red algal group

After filtering, the number of genes actually used this analysis of each species is shown in Supplementary Table S8. The codon bias of different regions of all genes in three categories is shown in Fig. 6 and Supplementary Table S8. For the high-bias genes category, Fop of the domain was markedly higher than that of the nondomain in 6 species (*C. merolae, P. purpureum*, *C. crispus, G. chorda, G. domingensis* and *P. umbilicalis*) and for the medium-bias gene category, Fop of the domain was markedly higher than that of the nondomain in seven species(except for *G. sulphuraria*), but for the low-bias gene category, only 2 species showed Fop values of in the domain that were markedly higher than those of the nondomain(*C. crispus* and *P. umbilicalis*). This phenomenon is consistent with the accuracy of translation selection, which is an important driving factor of codon usage in red algae, and it is more apparent in the high-bias category and the medium-bias category and even found in the low-bias category of individual species.

### Estimation of translation selection strength in eight red algae

Analyses of all eight genomes showed that natural selection plays an important role in translation accuracy. Researchers have designed many methods to determine the selection strength of codon usage. Here two different methods were used to estimate the strength of natural selection in Rhodophyta.

Assuming that the codon bias is in a mutation-drift-selection equilibrium, the odds ratio was used to estimate the translational selection strength in enterobacteria [34]. The odds ratios of nine twofold degenerate amino acids from eight red algae are shown in Supplementary Table S9, which reveals that except for Phe in *C. yangmingshanensis*, the odds ratios ranged from 1 to 11, which were similar to those in enterobacteria [34], holozoan protists [35], *Drosophila melanogaster* [36] and *Arabidopsis thaliana* [36].

Based on the population genetic model developed by Bulmer [24], widespread research on the codon usage of 80 bacterial genomes was performed to gain the most important statistical parameter, S (the log of the odds ratio) [22], which was used to estimate the translation selection strength. dos Reis, et al. [36] reported that *Ŝ*, the weighted average value of S in each genome can be used to accurately estimate the codon selection strength in eukaryotes. The S and *Ŝ* of eight red algae are shown in Supplementary Table S9 and Table 1, in which S ranged from 0 to 3 and *Ŝ* ranged from 0.6 to 1.5. Except for Phe, the S of other amino acids were greater than 1 in *P. purpureum*, *C. crispus* and *P. umbilicalis.* For the nine twofold degenerate amino acids, the S of 6 amino acids was more than 1 in *G. sulphuraria*, but S was less than 1 in the remaining species. Corresponding to S, Ŝ was greater than 1 in *P. purpureum, C. crispus* and *P. umbilicalis*, and the Ŝ for *P. purpureum* was similar to that for *Monosiga brevicollis,* and those for *P. umbilicalis* and *C. crispus* were slightly higher than those for *Monosiga brevicollis* [35]. The Ŝ for *G. sulphuraria* was calculated to be 0.97, which is similar to that for *Drosophila melanogaster* [36]. The Ŝ values of the other four species are comparable to those of some diatoms [14]. Previous studies have shown that there is selectivity in the codon usage of *Monosiga brevicollis*, *Drosophila melanogaster* and diatoms [14, 35, 36]. Therefore, there is also selection pressure on the codon usage of eight red algal genomes.

## Discussion

Our analysis revealed similarities and differences in CUB in red algae. Most red algae prefer codons ending with CG, and especially in *P. umbilicalis*, the codons exhibit very strong GC bias (Table 1). The value is similar to the GC3 values of some species(0.70–0.86) in Chlorophyta reported by Li*, *et al*.* [37]. *P. purpureum* also has a strong bias for GC3, with values similar to the GC3 of *Volvox carteri*(0.697) in Chlorophyta and some species(0.6–0.63) in Monocotyledons [37]. *C. yangmingshanensis, C. merolae* and *C. crispus* showed a marginal preference for codons ending with GC, similar to the GC3 of *Selaginella moellendorffii* (0.593) in Pteridophyta and most species(0.57–0.63) in Monocotyledons [37]. *G. chorda* and *G. domingensis* show an almost neutral preference for four codons, with values similar to the GC3 of *Physcomitrella patens* (0.5) in Bryophyta, *Musa acuminate* in Monocotyledons (0.528), *Linum usitatissimum*(0.501) and *Eucalyptus grandis*(0.522) in Dicotyledons [37]. *G. sulphuraria* is different from other species and showed a strong preference for codons ending with AT, which is similar to the findings in *Picea abies*(0.438) [37], *Pinus taeda*(0.428) [37] as well as *Taxus contorta* (0.401) [38] of Gymnosperms, and most species (0.355–0.486) of Dicotyledons. Overall, in the eight red algae, as the ancestors of green plants, show a large degree of variation in the GC3 content of genes of the nuclear genomes during the evolution, in which *P. umbilicalis* is similar to Chlorophyta, *G. chorda* and *G. domingensis* are similar to Bryophyta, *C. yangmingshanensis, C. merolae* and *C. crispus* are similar to Pteridophyta, *G. sulphuraria* is similar to gymnosperms, *P. purpureum* is similar to Monocotyledons, and *G. sulphuraria* is similar to Eudicotyledons.

In addition, among the 7 species of red algae, the frequency of using codons ending with CG is significantly higher than that of AU, which is similar to some species in Chlorophyta, Pteridophyta and Monocotyledons [37]. The frequency of using codons ending with AU is significantly higher in *G. sulphuraria*, which is similar to observations in some species of Dicotyledons, Bryophyta and Gymnosperms [37]. Schonknecht*, *et al*.* [39] speculate that *G. sulphuraria* might have obtained a large number of AT-rich genes from extreme thermophilic bacteria and archaea through gene transfer.

Codon usage bias is gradually formed in various organisms mainly through selection, mutation and drift [20, 24]. Comparing plastid genomes [27, 28], the effects of mutations and drift on codon bias are limited in the nuclear genomes of red algae, and selection plays a dominant role in the formation of codon preference, especially in high-bias genes, which affects CUB by improving translation efficiency and translation accuracy. First, in our research, the Fop, CAI and CBI values of high-bias genes were higher than those of medium- and low-bias genes (Supplementary Table S10), consistent with findings in *Escherichia coli* [40], which indicates that red algae improve translation efficiency by using more optimal codons in high-bias genes. Second, in high- and medium-bias genes and even in individual low-bias genes of most species, the Fop values in the structural domain were greater than those in the nondomain (Fig. 6), in accordance with the results for Holozoan protists [35] and *Escherichia coli* [40], which indicates that red algae improve translation accuracy by using more optimal codons in the protein domain (conserved sites).

Interestingly, many tRNA modifications were found in the highly degenerate amino acids of all multicellular red algae and individual unicellular red algae in our research, which appear in Holozoan protists [35], most plants [41] and animals [42–44] by tRNA deamination under ADAT [41, 45–47].

Some studies have shown that transgenic design using optimal codons can improve the level of heterologous gene expression [48–50]. Some of the red algae studied here have important ecological, edible, medicinal, ecological, industrial and other value [51, 52], and red algae are also important materials for studying the origin and evolution of green plants. Our data provide important reference information for the successful expression of artificial transgenes. In transgenic engineering, using the optimal codon of a species can maximize the production of its encoded protein, increasing it by three orders of magnitude [53]. Table 2 shows the optimal codons and optimal termination codons of the eight species, which will make it possible to successfully apply transgenic design for each red alga in the future.

## Conclusion

Overall, by studying the codon usage patterns of *C. merolae, G. sulphuraria, C. yangmingshanensis, C. crispus, G. chorda, G. domingensis, P. purpureum* and *P. umbilicalis*, it was found that high-bias genes in green plant ancestors show a preference for codons ending with GC. Correlation analysis indicated that the codon bias pattern was significantly correlated with Fop, the CAI and the CBI. Nc-GC3s plot analysis showed that the codon bias pattern of most genes is affected by selection because of deviation from the expected curve. Neutrality plot analysis indicates that natural selection plays a dominant role in the formation of codon usage patterns, which was also proven by comparing protein domain and nondomain codons. The analysis comparing the nucleotide content of intron and flanking sequences showed that natural selection strongly affects the codon usage pattern by driving the high GC content of high-bias genes, especially in unicellular species. Finally, we have obtained 15 common optimal codons in the seven red algae except for *G. sulphuraria* and the characteristics of related tRNA genes. Our results lay a foundation studying transgenic expression of economic red algae and the evolutionary study of codon usage characteristics of green plant ancestors.

## Materials and methods

### Data source

To analyze the codon usage pattern of green plant ancestors, the genome and annotated files of all red algae were downloaded from the NCBI Genome database, including four unicellular Rhodophyta (*Cyanidioschyzon merolae, Cyanidiococcus yangmingshanensis, Galdieria sulphuraria* and *Porphyridium purpureum*) and four multicellular Rhodophyta (*Chondrus crispus, Gracilariopsis chorda, Gracilaria domingensis* and *Porphyra umbilicalis*). The versions of the genome and its annotation file from the eight species are shown in Supplementary Table S11. By using TBtools software, we obtained the coding sequences of these species [54]. To reduce sampling error, we screened the sequences based on the following considerations: (1) the length of each CDS was a multiple of three; (2) ATG was the start codon, and UAA, UAG or UGA was the stop codon, (3) there was no termination codon in the middle of the CDS (Excluding the coding sequences with a premature termination); (4) the length of the sequence was greater than 300 bp; (5) repetitive sequences were removed, and the longest transcript was selected; (6) pseudogenes were eliminated; and (7) sequences with poor sequencing quality were excluded.

### Codon usage analysis

Codon W 1.4.2 [55] was used to calculate the GC, GC1, GC2, GC3, Nc, CAI, CBI, FOP and RSCU values of each species and SPSS Statistics 27 was used to perform the correlation analysis among these parameters. According to the order of Nc values, the 5–10% of genes with the lowest values were chosen as the high-bias gene category (high-expression gene category), the 5–10% of genes with the highest values were chosen as the low-bias gene category (low-expression gene category), and the rest with the middle values were chosen as the medium-bias gene category. The detailed percentages for each species were as follows: 5% (*C. merolae*), 7% (*C. yangmingshanensis*), 5% (*G. sulphuraria*), 5% (*P. purpureum*), 8% (*C. crispus*), 9% (*G. domingensis*), 10% (*G. chorda*) and 5% (*P. umbilicalis*). The codons with an the RSCU difference between the high-expression gene category and low-expression gene category greater than 0.08 was defined as the optimal codons [56].

### Defining the GC content of noncoding regions

The introns and flanking DNA of genes were extracted and screened from the genome and its annotation files according to the following rule. If conditions permit, we can extract 200 bp flanking DNA from each 5’ and 3’ end of each gene. When the intergenic region was < 200 bp, or a gene was located at the end of a contig or a scaffold, we extracted the maximum possible length of flanking DNA [35]. We extracted and connected all introns of each gene to calculate their GC content through Codon W, which is similar to the flanking DNA. Finally, the average value and standard deviation of each category were calculated.

### Nc-GC3s plot analysis

Nc-GC3s plot analysis was used to explore the relationship between GC3s and Nc values [21]. In the rectangular coordinate system, the GC3s values of each gene were defined as the abscissa, and the Nc values were defined as the ordinate. After that, we drew an expected gene curve, which represented the expected position of genes only under neutral mutation pressure. Finally, we confirmed the effects of neutral mutations and natural selection on the codon bias of species through a comparison of expected gene values and actual values [21].

### Neutrality plot analysis

Neutrality plot analysis was used to assess the effects of different drivers on codon bias. In the plot, the GC12(average of GC1 and GC2) of each gene was regarded as the x-axis and GC3 as the y-axis, establishing a rectangular coordinate system for regression analysis. The slope of the regression curve reflected the degree of influence of different drivers, mutation pressure or natural selection. When the slope was close to 0, most of the influence was natural selection [57].

### Parity rule 2 plot

Using MEGA X software, we calculated the values of A3, T3, C3 and G3 for all genes of each species, regarding G3/(G3 + C3) and A3/(A3 + T3) as the abscissa and ordinate, respectively. The influence of mutation on codon usage bias can be inferred from the proportion of four bases in the figure. If preference was affected only by mutation, then the frequency of bases A/T and C/G at codon 3 would be similar, that is, A≈T or G≈C; otherwise it may be affected by other factors such as natural selection [58, 59].

### Prediction and screening of tRNA genes

The downloaded genome sequence files of 8 red algae were scanned using the default parameters of tRNAscan-SE v.2.0.9 software to predict tRNA genes [60].

### Frequency of optimal codon usage for domain and nondomain codons

To determine whether codon bias affects different regions of genes in red algae, the frequency of optimal codons (Fop) was measured and compared between the domain and nondomain regions of the three bias categories. First, each gene was divided into putative functional domain and nondomain codons according to the NCBI's Preserved Domain Database (CDD). However, according to the method of Southworth [35], the following genes were excluded: (1) genes that did not code annotation regions were excluded from the analysis; (2) when the functional domain spanned all codons of the whole gene, the gene was excluded; and (3) when the functional domain spanned all codons except for the start codon and/or the stop codon, the gene was excluded. Second, the values of Fop for the domain and nondomain regions were determined by Codon W. Because some domain and nondomain regions may be too short to calculate the value of Nc, Fop was used to determine the codon use bias of the regions [35].

### Estimation of the strength of translational selection

The strength of translational selection was estimated by using the following two methods according to Eyre-Walker*, *et al*.* [34] and dos Reis*, *et al*.* [36]. First, the highly expressed gene category and lowly expressed gene category were determined. Then, the optimal codon and the suboptimal codon were identified for both categories. Finally, the parameters of translation selection strength, odds ratios and S were calculated. The odds ratio can be calculated by calculating the relative difference of 2 degenerate synonymous codons between the highly expressed gene category and the lowly expressed gene category (Formula 1). S was the logarithm of the odds ratio used to estimate strength of translational selection [22]. Using this method, the odds ratio and the S of eight red algae were calculated [34].

1 $$\text{odds ratio }=\frac{\text{f}1H}{\text{f}2H} *\frac{\text{f}2L}{\text{f}1H}$$

In the above formula, f represents the codon frequency. The subscripts 1 and 2 represent the optimal codon and suboptimal codon respectively; the subscripts H and L represent the high-bias gene category and the low-bias gene category, respectively.

By the second method from dos Reis, the *Ŝ* of the eight genomes was determined by calculating the weighted average of S for the number of codons of the nine 2-degenerate amino acids in the high-bias gene category to estimate the codon usage bias strength of the species genomes[22, 34, 36].

### Supplementary Information

**Additional file 1:** **Supplementary** **Table S1.** GC3s of CDSs, GC content of flanking sequences and introns respectively in the three bias categories from the eight red algae.

**Additional file 2:** **Supplementary** **Table S2.** Correlation analysis and t test (double tail) for the GC3s of CDSs and GC content of introns among the eight red algae.

**Additional file 3:** **Supplementary** **Table S3.** Output of optimal codon analysis from Codon W in the eight red algae (S3a-S3h).

**Additional file 4:** **Supplementary Table S4.** Output of tRNAscan-SE from whole genome contigs in the eight red algae (S4a-S4h).

**Additional file 5:** **Supplementary** **Table S5.** The optimal codon which lack perfectly matching complementary tRNA genes in multicellular red algae.

**Additional file 6:** **Supplementary** **Table S6.** Number of tRNAs and anticodons of different amino acids in the eight red algae (S6a-S6h).

**Additional file 7:** **Supplementary** **Table S7.** Correlation analysis and significance test (t test, double tail) for 12 parameters related to codon usage among eight red algae (S7a-S7h).

**Additional file 8:** **Supplementary Table S8.** The average Fop values for the domain and nondomain codons of the three bias gene categories in the eight red algae.

**Additional file 9:** **Supplementary Table S9.** Odds ratios and their logarithmic values(Ln) of two degenerate amino acids among the eight red algae.

**Additional file 10:** **Supplementary** **Table S10.** Mean and standard deviation of CAI, CBI, and Fop for each bias category of the seven red algae (excepting for Galdieria sulphuraria).

**Additional file 11:** **Supplementary** **Table S11.** Versions of genomes and annotation files and taxonomic information for the eight red algae.

**Additional file 12:** **Supplementary** **Figure S1.** The plotting for GC3s of CDSs and GC content of introns among eight species. The correlation between two statistical data points is represented by the plotting of the GC3s of CDSs and GC content of introns in the same gene. The black solid line represents the correlation line, and its equation is shown at the top of the plot.

## Data Availability

The taxonomic information and genomes data that support the findings of this study are openly available in [NCBI] at https://www.ncbi.nlm.nih.gov/. The remaining data generated or analyzed during this study are available within the article and its supplementary materials.
