# Supplementary material for: Codon usage pattern of the ancestor of green plants revealed through Rhodophyta
Source: BMC Genomics. 2023 Sep 11;24:538. doi: 10.1186/s12864-023-09586-w (PMC10496412; doi:10.1186/s12864-023-09586-w)
Supplement: Supplementary file 12 — Additional file 12: Supplementary Figure S1. The plotting for GC3s of CDSs and GC content of introns among eight species. The correlation between two statistical data points is represented by the plotting of the GC3s of CDSs and GC content of introns in the same gene. The black solid line represents the correlation line, and its equation is shown at the top of the plot. [file 12864_2023_9586_MOESM12_ESM.docx]

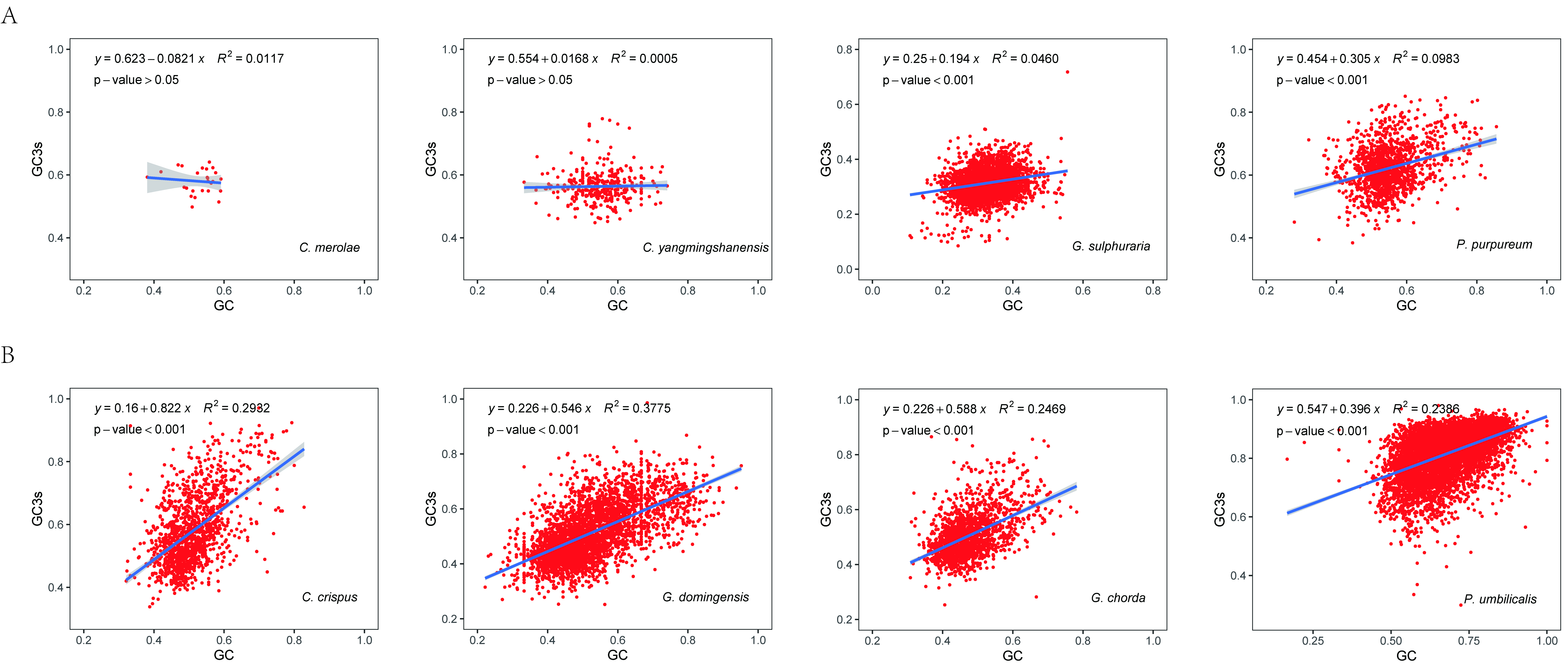


**Figure S1**: The plotting for GC3s of CDSs and GC content of introns among eight species. The correlation between two statistical data points is represented by the plotting of the GC3s of CDSs and GC content of introns in the same gene. The black solid line represents the correlation line, and its equation is shown at the top of the plot.
